# Improved chaotic Bat algorithm for optimal coordinated tuning of power system stabilizers for multimachine power system

**DOI:** 10.1038/s41598-024-65101-5

**Published:** 2024-07-02

**Authors:** Mohammed Tadj, Lakhdar Chaib, Abdelghani Choucha, Mohannad Alhazmi, Abdullah Alwabli, Mohit Bajaj, Shir Ahmad Dost Mohammadi

**Affiliations:** 1 Energy and Materials Laboratory, University of Tamanghasset, Tamanghasset, Algeria; 2 Electrical Engineering Department, College of Applied Engineering, King Saud University, P.O. Box 2454, 11451 Riyadh, Saudi Arabia; 3 Department of Electrical Engineering, College of Engineering and Computing in Al-Qunfudhah, Umm Al-Qura University, Mecca, Saudi Arabia; 4 Department of Electrical Engineering, Graphic Era (Deemed to be University), Dehradun, 248002 India; 5 Hourani Center for Applied Scientific Research, Al-Ahliyya Amman University, Amman, Jordan; 6 Graphic Era Hill University, Dehradun, 248002 India; 7 Department of Electrical and Electronics, Faculty of Engineering, Alberoni University, Golbahar, Kapisa Afghanistan

**Keywords:** Chaotic maps, Chaotic NBA (CNBA), Multimachine power system, Novel Bat algorithm (NBA), Power system stability, Power system stabilizer, Energy science and technology, Engineering, Mathematics and computing

## Abstract

Power systems exhibit nonlinearity. causing dynamic instability and complex power oscillations. This research proposes an innovative strategy using the Novel Bat Algorithm (NBA) to achieve ideal Power System Stabilizers (PSSs) in a multimachine power system. The approach shifts electromechanical modes to specific areas in the s-plane. Enhancing the multi-machine power system and establishing stabilizer parameters for dynamic performance. The study examines the designed approach aptitude for standard lead-lag PSSs configurations. In order to elevate the global search problem and transfer some static operators for the optimum optimization process. the chaos mapping. also known as CNBA. is introduced into NBA. Four different forms of chaos maps are compared in experiments to resolve unconstrained mathematical issues in order to illustrate CNBA performance. In any other case. the challenge of designing PSS under a wide range of loading situations is transformed into an optimization challenge with the damping ratio of electromechanical modes with low damping as the target function. The optimal stabilizers’ gains are gotten by employing the CNBA algorithm. Second plan. an effective technique is astutely established to delineate the PSS location and quantity using CNBA and another side using participation factor. To examine the efficacy of the proposed CNBA-based PSS on a large system; it is tested on the interconnected of New-England/New-York (16 generators and 68 buses) power grid. and verified by comparative study with NBA through eigenvalue analysis and nonlinear simulation to provide evidence the algorithmic competence of CNBA. The CNBA approach yields a minimum damping ratio of 37%. which is consistent with the its eigenvalue. In contrast, the NBA approach achieves a minimum damping ratio of 31%. The simulation results reveal the fine performance of the proposed CNBA-PSS in a convincing manner and its capacity to provide an excellent damping for inter-area and local oscillations under diverse operating cases compared to NBA-PSS then in the case of PSS location.

## Introduction

### Motivation and incitement

**Figure 1:**
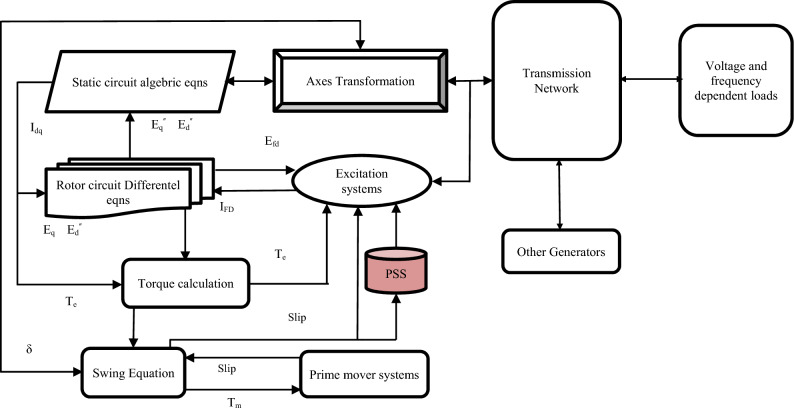
Multimachine power system scheme.

In the early years of power systems. there was not much development in power generation. Power systems inherently exhibit nonlinearity and experience various transient conditions. resulting in dynamic instability and power oscillations that are complex to control. Small signal perturbations noticed on the power system are caused by various contingencies such as generator outages and heavy power transmitted over weak tie-line^1^. The Flexible Alternating Current Transmission System (FACTS) is a reliable component based on power electronics. which offers an occasion to enhance stability. power transfer capability of AC transmission systems. and adjustability. Also. it ensures a deep analysis of the development and enhancement in the power system stability progress^2^. Guaranteeing power system stability represents one of the elementary practical difficulties in power engineering. It must get a position in the concept and expansion phase of a power system. Regulation systems such as PSSs depict supplementary elements. i.e. means of enhancing mitigating transient states and stability. Synchronous generators denote the leading power in power systems. which are provided with additional damping circuits that generate moderately elevated electromagnetic damping torques. Nevertheless. the excitation systems operation. specifically rapid unchanging ones. can diminish the torque values. unfavorably influencing the wave shape of electromechanical temporary states. This disapproving effect of voltage regulation systems could be diminished. including others. by the utilization of supplementary. adaptable elements called Power System Stabilizer (PSSs). comprehended as standard systems^3^. Then. an important feature for ensuring the security of a multimachine power system is the damping of low-frequency oscillations (LFOs). Consequently. PSS has often been employed to mitigate the electromechanical oscillation modes of the generators in large power systems. The traditional PSS is typically developed using a linear model of the system for a specific operating condition. The main challenge of PSS designing is according to their parameters that must be able to remain the power system to its limit after being subject to fault^4^. Multimachine power system scheme is shown in Fig. 1.

### Literature review

The researchers have investigated various approaches. surrounding classical. deterministic. and metaheuristic algorithms. to assurance the precise evaluation of power system stability. It must be highlighted that the employment of conventional PSSs in the advanced numerical voltage controllers to regulate the synchronous generators does not need enormous financial costs^4^. Consequently. a PSS is applied as a supplementary portion in the voltage controller package contrasted to other clarifications enhancing the power system stability. It represents merit remarking that the enhancement in the case of the damping of electromechanical temporary states of power systems appreciates the application of PSSs fixed in excitation systems and declines the property of voltage regulation^5^. However. the influences of standard PSSs could be equivalent to the other systems' influences. supplied that they are suitably nominated. Subsequently. the scientific research and utilization of standard PSS are even confirmed.

Presently. numerous types of stabilizers are exercised in power systems. beginning with the basic individual input until complex. broadband. and multi-input ones. The individual input stabilizers (PSS1A-type) are modest in structure and adjusting but have their disadvantages; the PSS which have individual input from rotating speed in the case when the speed is determined just in a single position of the generator axe could augment the torsional oscillation of the generating section. Adjusting PSSs represents more complexity. It denotes correspondingly significance highlighting that inaccurately chosen PSS parameters could be deteriorated. not enhanced^3^.

Authors in^6^ suggest an adaptive fractional fuzzy sliding mode controller based-PSS for damping out low-frequency fluctuations in multimachine and single machine infinite bus under different operative unforeseen. Swain et al. in^7^ present an adaptive interval type 2 fuzzy sliding mode controlled-PSS to disable the low-frequency electromechanical oscillations and improve stability under suspicions and peripheral oscillations. In^8^ the work proposes a process for adjusting SMC gains for a PSS utilizing a deep neural network. This regulator needs rapid switching which could produce undesirable signals. To explain this difficulty. a limit layer is exercised.

In^9^ researchers offer a linear quadratic Gaussian (LQG) on PSS to regulate the subsynchronous resonance (SSR) that could appear in a Series Capacitor Compensated Power System (SCCPS). It chooses the concept parameters to approve the stability robustness and examines the principal parameters of the SSR applying the critical compensation level (CCL). Yathisha et al. in^10^ propose a collection of highly improved Unifilaire Power Flow Controller (UPFC) to regulate input lengthways with an instantaneous synchronized strategy of PSS which represents a challenge in power system. The experiment settings are applied with no including oscillations case where switching is completed exercising Linear Quadratic Regulator (LQR). The second setting is applied with oscillations employing LQG. The authors in^11^ suggest a control method to enhance the damping ability of sub-generator fluctuations through the regulation of a LQR to decrease the oscillations in the power system. The proposed model includes the coordination of the PSS in conjunction with LQR regulators.

For solving the mathematical optimization approach. the design of the problem must assure mathematical constraints with complex computer algorithm conditions. Recent advances in computer technology have made it possible to use optimization tools to aid in the implementation of control and to treat the problems of the real-world. Further. as several real-world optimization problems turn out to be increasingly difficult. superior optimization tools are constantly needed. The conventional optimization algorithms. including the simplex and the gradient descent approaches. may not provide satisfactory solutions as they require the gradient information of the objective function (OF) and may get stuck in a local minimum of highly intricate functions. Newly, flexible metaheuristic optimization algorithms have been implemented for solving problems with high dimensions. In recent decades. modern optimization algorithms have emerged as effective tools for treating complex optimization troubles. Among these algorithms, Genetic Algorithm (GA), Particle Swarm Optimization (PSO), Differential Evolution (DE), Evolutionary Algorithm (EA), Bat algorithm (BA), and others have been widely applied in various fields, including power system optimization. In particular. several studies have focused on designing PSS using these algorithms to enhance power system reliability. Below state of art has been made for this subject^12–14^.

GA has been extensively employed as an optimization tool for PSS parameter tuning in multimachine power systems. Many papers in the literature have proposed GA-based techniques to optimize the PSS parameters for a broad limit of operating conditions. These papers have proposed different approaches to improve the performance of the power system. such as simultaneous coordination of multiple PSS. optimal location and parameter tuning of PSS. and simultaneous stabilization of multimachine power systems over several loading conditions.

The GA application for the simultaneous stabilization of multimachine power systems via single-setting PSS over a broad limit of operating conditions is presented in^15^. The PSS parameters selection. which can stabilize the power system. is transformed into an optimization problem that is solved using GA with an eigenvalue-based OF. The effectiveness of simultaneous stabilization is validated by considering different loading conditions in a multimachine power system. In^16^. a method for determining the optimal number and location of PSS in a multimachine is proposed using participation factor (PF) and GA. The PF technique is used to estimate the number of PSS and recognize their location. while GA is employed to further decrease the PSS number and optimize their location and parameters for several operating points. The design problem is done as an optimization with multi-objective problem that involves increasing the damping ratio of the electromechanical modes. The use of GA for tuning PSS parameters has been proposed in^17^. which involves dynamically adjusting the search space limit through the optimization process in order to explore promising regions that could potentially hold the global optimum. This adaptive space of search permits the GA to generate novel solutions that would not be possible with a fixed space of search. leading to a more diverse population. The proposed algorithm has been tested on a multimachine power system.

In^18^ a design and implementation of PSS for a multimachine power system is proposed using an innovative evolutionary algorithm. namely Breeder GA with Adaptive Mutation. To analyze the effectiveness of the proposed approach. a PSS was designed and implemented on a weak connected three-machine test system. The work given in^19^ proposes a GA that simultaneously adjusts multiple PSS. PSS designing is supposed to be unvaried including essentially lead-lag filters. The adjustment technique is designed to ensure system stabilization under various operating conditions. taking into account its effectiveness. Enhanced GA operators are employed in the optimization of various PSS parameters.

In^20^ a simultaneous coordination of multiple power PSS using GA is proposed. where both local and remote measurements are the input signals to the PSS. The GA algorithm is employed to tune the parameters of both single and multiple PSS. and the suggested design is verified on a multimachine power system to demonstrate its effectiveness. The use of a hybrid technique that combines GA and gradient method for tuning power system stabilizers is done in^21^. This method. named GA-GR. aims to dampen power system oscillations by optimizing the damping factor and the real part of eigenvalues as a multi-OFs. The proposed coordinated and robust tuning procedure is demonstrated on a range of power system models. In^1^ an objective based on changed and inconstant weight factors under eigenvalues form is planned and optimization parameters tuning of PSS is performed via GA. The influence and powerful synchronization monitoring through dual-fed induction generator and synchronous machines with power system stability improvement and PSS. on the crucial LFOs. The tunning of PSS is employed using GA. The non-imaginary part and damping coefficient of eigenvalue variation are selected as fitness function and presented in^2^. Recently, many authors have effectively used PSS in power system network to improve its stability^22–26^.

PSO is a popular metaheuristic optimization algorithm that has been employed to tune the parameters of PSS in many papers. particularly for multimachine systems. Its ability to quickly converge to the global optimum solution and handle non-linear and non-convex problems makes it a popular choice for power system stability improvement. In^27^ the authors implemented three different types of PSO-based PSS for improving dynamic stability in a power pool. Three different systems are investigated with each initially operating as a standalone system. To improve system stability. a PSS is designed and installed in each power system using one of three PSO variants. Eslami et al. in^28^ present an approach to determine the PSS optimal location using the integrating PSO with the chaotic. The change in the PSO algorithm is completed by including passive congregation which represents a good biological potential that conserves swarm integrity. Authors in^29^ propose in the multi-machine power system an instantaneous coordinated Static VAR Compensator (SVC) and scheming of PSS as a damping stabilizer. PSO and chaos concept are combined to produce a chaotic PSO (CPSO). which practically associates the population-based evolutionary searching ability of chaotic searching comportment and the PSO method.

In^30^ a crow search algorithm is proposed for the optimal design of PSS in a multimachine power system. The suggested algorithm is verified on a multimachine power system and its efficiency is evaluated. The authors in^31^ have treated the problem of PSS parameters coordinated tuning of a small power system. The design problem is converted to an optimization problem. The optimal tuning of a PSS is obtained using PSO. In^32^ a robust method for the optimal adjustment of PSS is presented. by inserting the improved PSO with chaos (MPSOC). The modification in PSO is achieved by inserting Passive Congregation (PC). which allows each swarm member to receive information from multiple other members and thus it reduces the likelihood of a detection failure or a fruitless search. Additionally. MPSOC is used to enhance the capability of global search and avoid premature convergence due to local minima. The proposed algorithm is employed for optimal PSS parameter tuning and tested on a multi-machine power system. In^33^ Two conventional bio-inspired methods: bacterial foraging algorithm (BFA). and the small-population-based particle swarm optimization (SPPSO). are suggested for the Synchronous concept of multiple best PSSs in two power system.

DE has been extensively applied to treat the optimization problems in power system stability research. including the design and tuning of PSS. In^34^ a robust conventional scheme for improving power system stability is proposed. which involves designing multiple and multi-type damping PSS that use local measurements as input signals. The coordinated design trouble is considered an optimization problem. which is solved using the DE algorithm to attain the optimal PSS parameters. The effectiveness of the stabilizer is demonstrated for both the single-machine and multi-machine power systems. In^35^ the paper addresses the optimal adjustment of a multi-band PSS for enhancing the damping of power oscillation. The authors convert the optimization problem into a quadratic criterion minimization. which is treated using the DE optimizer. To reduce problem dimensionality and complexity. dynamic equivalents are employed. The proposed approach is tested on the high-voltage Mexican power grid.

The problem formulation is a linear controllers problem where the DE algorithm is applied to adjust the designed PSS parameters. The Dual Input PSS (DIPSS) is treated in^36^ to attain an additional signal to the excitation system in order to generate suitable flux in case of a perturbation occurring. DIPSS is employed to treat the disturbance and directly offer a suppressing to the perturbation source. The DIPSS controller parameters are optimally achieved through the DE algorithm.

Newly. a robust metaheuristic algorithm has been elaborated which is called Bat algorithm (BA) by Xin-She Yang^37^. The algorithm is based on the echolocation behavior of microbats and has shown promising results in preliminary studies. Compared to other well-known metaheuristic algorithms like GA and PSO. BA has demonstrated superior performance^37,38^. The paper presented in^39^ used BA to optimize the parameters of gain and pole-zero of a PSS for a small power system model. The optimization is achieved by defining fitness amount via eigenvalue shifting to ensure the stability of the nonlinear plant across a broad limit of operating points. On the other hand. In^40^ the BA is proposed for the optimal design of PSS in a multimachine power system. The problem of PSS parameter tuning is treated as an optimization trouble and solved using the BA. Also in^41^ a novel PSS structure is investigated for the first time through the BA to enhance the stability of the power system. The problem formulation has been adopted in^42^ as a probabilistic and deterministic tuning issue of synchronizing several PSS in order to enhance the power system stability. The directional Bat algorithm is chosen to solve and tune PSS parameters.

In recent years. numerous studies have been conducted to apply these novel algorithms to power system stability improvement. Researchers have proposed various metaheuristic algorithms for PSS tuning. Each of these algorithms has unique features that make them suitable for certain types of problems. By applying these algorithms to PSS tuning. researchers have been able to obtain optimal PSS parameters that improve the stability of power systems. The effectiveness of these algorithms has been demonstrated in many papers. Some of the studies have tested the algorithms on different types of power systems with varying operating conditions and perturbations. By optimizing the PSS parameters. researchers have been able to reduce the oscillations in power systems. increase the damping ratio. and improve the dynamic stability of the system.

In^43^ a method for optimally adjusting PSS parameters in a multi-machine power system is shown using population-based incremental learning that hybrids aspects of GA and competitive learning based on artificial neural networks. The method uses a probability vector to generate better individuals and is considered to be easy. transparent. and efficient with respect to problem illustration. In^44^ a multi-objective approach called parallel vector evaluated improved honey bee mating optimization is introduced to obtain optimal PSS parameters for a multimachine power system. The approach uses two fitness functions: eigenvalues based on the damping factor and time-domain errors. It considers a remote generator’s feedback signal and remote input signal ratios for each generator in the system and treats the tuning problem as a multi-objective optimization process.

In^45^ a comparison of three metaheuristic optimization algorithms is done for PSS tuning in a multimachine power system. The algorithms are ACO. BA. and GA. The optimization problem is to minimize system oscillations under a set of pre-specified operating conditions. The PSS parameters are adjusted using the designed methodologies in the test system. The article^46^ proposes an optimal PSS design for dynamic stability in a multimachine power system using the Cuckoo Search (CS) algorithm. The tuning problem is structured as an optimization problem based on a damping ratio. and the CS algorithm is used to solve it. The study is simulated under various operating conditions and perturbations. The research described in^47^ addresses the problem of designing PSS for multimachine systems and introduces the chaotic teaching–learning algorithm (CTLA) to ensure a global search. A chaotic phase is included in the CTLA to improve its effectiveness. The proposed algorithm is evaluated on a multimachine system to demonstrate its efficacy in power systems.

In^48^ the authors proposed a novel structure for a multi-band PSS. in which the gains are optimized using a novel meta-heuristic algorithm. The algorithm is an arrangement of PSO. culture algorithm. and co-evolutionary algorithms. and is referred to as the Culture-PSO-co-evolutionary (CPCE) algorithm. The effectiveness of the CPCE algorithm is verified in a multimachine power system. In^49^ the researchers proposed a metaheuristic method to enhance the power system stability of a multi-machine system using PSS. They used the Cultural Algorithm (CA) to obtain optimal PSS parameters and compared its performance with other metaheuristic algorithms. The optimal design and parameters of PSS for a multimachine power system are achieved using Harris Hawk Optimizer in^50^. Devarapalli et al. in^51^ present a method of combined grey wolf optimizer and sine cosine algorithm (SCA) for adjusting the PSS parameters of the multi-machine power system. A multi-objective fitness contains the damping and eigenvalue parts. In^52^ a PID controller-based PSS is applied for system stability improvement. The PID parameters have been attained perfectly by diminishing an OF to stabilize the unstable eigenvalues parts. The problem tuning of PSS parameters is solved using the moth search algorithm. In^24^ the study suggests the PSS parameters adjustment adopted in the SMIB power system. To do so. A hybrid procedure applied by combining atom search optimizer with simulated annealing is employed.

Bayu et al. in^53^ suggest the technique of Ant lion optimization (ALO) to tune the dual input PSS parameters to dampen the LFO of a region in the Ethiopian power system. ALO is utilized to gain the lowest damping coefficient at several operating conditions. In^54^ a hybrid optimization technique by integrating the weighted mean of vectors (INFO) method with Gaussian bare-bones and chaotic-orthogonal-based learning approaches. for reaching the best PSS parameters employed in a SMIB system. Butti et al.^55^ offer a PSS design used in a changed SMIB power system. The eigenvalue optimization is taken as a fitness function applied in changed SMIB using a modified whale optimizer. In^56^ a hybrid algorithm which is called the gradient-based and gorilla troops optimizer is suggested. it is presented as a suitable implement for tuning the PSS parameters employed in the IEEE four-generator.

To efficiently report an extensive choice of engineering challenges. the employment of a comprehensive metaheuristic algorithms is crucial^57–59^. In the power system stability enhancement. the Gravitational Search Algorithm (GSA) is used in^60^ for instantaneous synchronized designing of the PSS and Thyristor Controlled Series Capacitor (TCSC) owing a damping regulator. Kar et al. in^61^ develop an Adapted Sine Cosine Algorithm (ASCA) that utilized it to concept parameters of the PSS and Static Synchronous Series Compensator (SSSC) based on Lead-Lag controllers for the reason to improve the power system stability. A fractional-order PID controller adjustment applying the Modified Grey Wolf Optimization Algorithm (MGWOA) is offered in^62^ for power system stability enhancement in a SMIB system.

Table 1 announces a comparative analysis for some of those methods.

**Table 1:** A comparative analysis of methods.

Ref	Method	PSS type	OF	Remarks
^63^	Cross-Gramian Model Reduction Approach	PSS	ITAE	The PSS installed in order to ameliorate the low-frequency damping modes of fluctuations
^64^	Farmland fertility algorithm FFA	PSS	Eigenvalue	The FFA based PSSs concept establish to converge quicker with minimum calculation cost to find best PSSs setting
^65^	Farmland fertility algorithm FFA	Neuro-fuzzy based PSS	Eigenvalue	The FFA-PSSs application with IPFC evaluates in coordinated operation
^66^	Real-coded genetic algorithm	Interval type-2 fuzzy based wide area PSS	ITSE	The fuzzy rules number determination in IT2 Fuzzy Controller is useful for the quick response
^67^	Self-Adaptive Learning Bat Algorithm	Fuzzy based PSS	ITAE	The membership functions of fuzzy controllers optimized based on the performance index
^68^	Revamped sine–cosine algorithm (RSCA)	PSS	Eigenvalue	The synchronization of RSCA in the application facilitate the tuning of the PSSs parameters
^69^	Gorilla troops with the gradient-based optimizers	PSS	Eigenvalue	The combination outperforms the PSS settings
^70^	Send Time Optimization STO	PSS	Eigenvalue	The optimization algorithm evaluated as multi-objective for the tuning problem

### Contribution to the work

This work introduces an enhancement to the NBA that incorporates a robust algorithm. The algorithm ensures the confident generation of parameters for PSS and their optimal coordination. In subsequent sections of the article. the mentioned novel algorithm is referred to as CNBA. Various chaos maps are tested and the most effective one is ultimately selected. Numerous operating scenarios are put forward along with comprehensive discussions and analyses. Comparative analyses are conducted to highlight the significance of the proposed CNBA-PSS control strategy by comparing it with standard NBA. Hence. the modified NBA can effectively address a wide range of power system issues and optimize controller gains. It is expected that effective methods like chaotic maps will achieve superior solutions for addressing the desired power system in various situations. Throughout this paper. the following notable features are highlighted in order to numerically address the potential of new methods. such as chaotic maps (CMs). to deliver superior solutions for small signal stability: (i) The improvement of the Novel Bat search algorithm (NBA) through the insertion of chaos to optimize PSS in a multimachine power system. (ii) The improvement is done by using CMs and moving some static operators for better optimization performance. resulting in a new algorithm called CNBA. (iii) Four types of CMs are compared to solve unconstrained mathematical problems to demonstrate CNBA's performance. and iv) The efficiency of the proposed CNBA-based PSS is verified and tested through nonlinear simulation and eigenvalue analysis. comparing it to NBA-PSS and using participation factor to locate PSS.

The organization of this work is partitioned as next. A summary of motivation and incitement. an introduction to the PSS controller. and the state-of-the-art of power system stability were reviewed in “Introduction” section. The modeling and design of the complete system is offered in “Power system modeling” section. The overview of the proposed algorithm with its improvements is presented in “Power system stabilizer” section. “Objective function” section demonstrates the results and discussions. Finally, the work is accomplished by a general conclusion in “Conclusion” section.

## Power system modeling

In this case. a model of fourth-order has been employed to model the synchronous machine. The formulated power system is given Eq. (1).as follows:

1 $$\dot X=f(X.U)$$

where $\dot X$ defines the vector of the state variables and $U$ denotes the vector of input variable. The state vector of n generators is given as ${\left[{\omega }_{i} {\delta }_{i} {E}_{qi}^{{{\prime}}} {E}_{fdi}\right]}^{T}$ and $U$ is the PSS output signal.

The PSS parameter value settings are often analyzed using this model^70,71^ as showen in Eq. (2).

2 $$\left\{\begin{array}{l} \dot \omega=\frac{\left({P}_{m}-{P}_{e}-D\omega \right)}{M}\\ \dot{{\delta }_{i}}={\omega }_{0}\left(\omega -1\right)\\ {\dot{E}}_{qi}^{\prime}=\frac{\left(-{E}_{q}+{E}_{fd}\right)}{{T}_{do}^{{{\prime}}}}\\ {\dot E_{fdi}} =\frac{-{E}_{fd}+{K}_{a}\left({V}_{ref}-{V}_{t}\right)}{{T}_{a}}\end{array}\right.$$

The power system's linearization model is utilized around its operating point. when examining dynamic stability. The power system's state equations can be expressed as follows in Eq. (3):

3 $$\dot X=AX+BU$$

where $A$ is a $4n\times 4n$ matrix and is given by $\partial f/\partial X$. while $B$ is the input matrix with order $4n\times m$ and is given by $\partial f/\partial U$. The $A$ and $B$ are calculated with each operating point. The state vector $X$ has order of $4n\times 1$ and the input vector $U$ has order of $m\times 1$. Thus. the multimachine power system scheme is illustrates in Fig. 1

**Figure 2:**
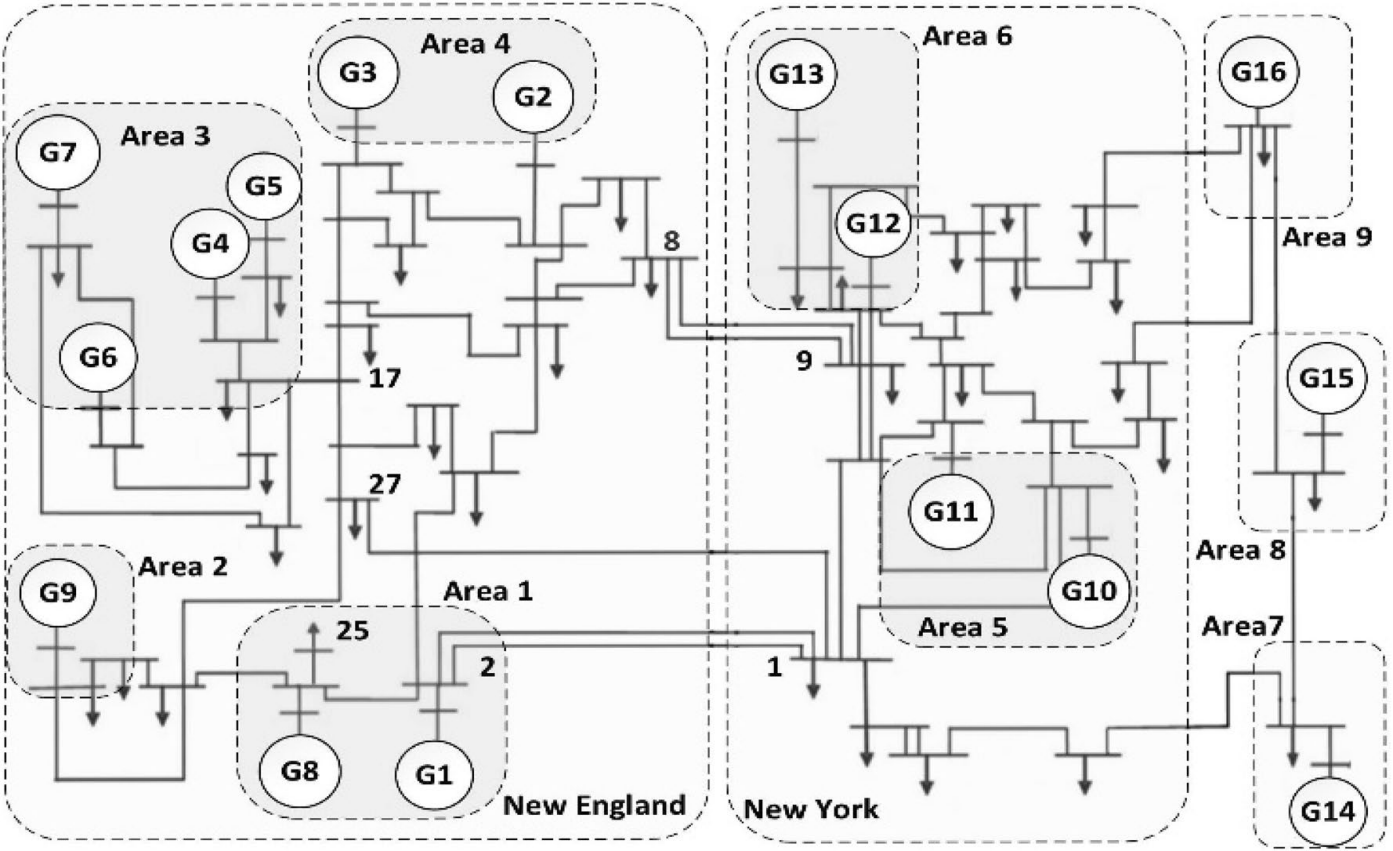
A sixteen-machine power system.

The designed scheme is evaluated by considering a multi-machine electric power system to assess its coherency. The benchmark model used in the study is the 16-machine 68-bus system. which takes into account a model of reduced order equivalent including in the New-England/New-York power system. On the other hand. generators G1 to G9 are present in the generation of the New England Test System, and the New York Power System (NYPS) has the generators G10 to G13 while generators G14 to G16 denote the dynamic equivalents of the three neighboring units that associated to the NYPS. Figure 2 shows the power system model while its system data can be found in^71^.

## Power system stabilizer

**Figure 3:**
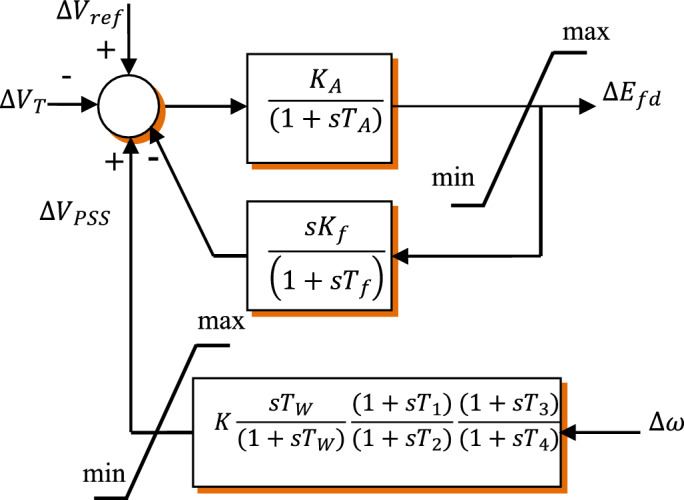
PSS with a lead-lag structure. in conjunction with an excitation system.

A PSS is a mechanism used in power systems to improve the damping of power system oscillations. These oscillations can occur due to various reasons such as sudden changes in system loads. disturbances in the grid. or changes in the generation capacity of the system. These unchecked oscillations can cause power system instability leading to system collapse. As shown in Fig. 3. The PSS employed in this study follows the conventional structure. and its transfer function. provided by Eq. (4)^72^ includes a gain block. suited via a high-pass filter of time constant. and blocks of lead-lag designed phase compensation with time constants. It should be noted that the aim of the suggested stabilizers is to improve power system stability by decreasing power system oscillations that occur after a significant disturbance. Usually, the input signal of such a design is the difference between the synchronous speed and the actual speed of the power system ${\Delta \upomega }$. and the output stabilizer $\Delta {\text{V}}_{\text{PSS}}$ represents a signal of voltage that is combined with the input signal of exciter system voltage. The transfer function can be depicted as follows:

4 $$\Delta {V}_{PSS}=K\frac{s{T}_{W}}{(1+s{T}_{W})}\left[\frac{(1+s{T}_{1})}{(1+s{T}_{2})}\frac{(1+s{T}_{3})}{(1+s{T}_{4})}\right]\Delta \omega$$

Profoundly, a PSS uses signals from the generator's excitation system to detect and damp out oscillations. It does this by injecting a supplementary control signal into the excitation system that is proportional to the generator's speed deviation. The supplementary control signal generated by the PSS helps to stabilize the generator's output voltage and frequency. As a result. the damping of oscillations will be improved. which enhances the overall stability of the power system. Figure 3. Illustrates a PSS with a lead-lag structure. in conjunction with an excitation system.

### Power system stabilizer types

#### Single input PSS

In rotor speed $\Delta \omega$ the inputs whose are determined to PSS are modified. the variation in the accelerating power ${\Delta P}_{a}$ and frequency $\Delta f$. In rotor speed the PSS1B has adjustment as input. This type of stabilizer will increase the damping of the low frequency oscillations. The PSS design shape for which the parameter namely lead lag time constants ${T}_{1}$. ${T}_{2}$ and ${K}_{C}$ gain. are to be quantified such when the PSS is involved in the feedback loop the global system becomes stable.

#### Dual input PSS (PSS2B)

One input as adjustment in rotor speed and separate input as adjustment in electric power. The variation in electric power ${\Delta P}_{e}$: Because of relationship to shaft speed and Uncomplicatedness of evaluating electric power. as a signal of input to PSS. To compute the $T$ and $K$ parameters the PSS employs the electric power as an input.

#### Multi band stabilizer (PSS4B)

The MB-PSS monitor the three frequencies scales. which are high. intermediate and low frequency scales. the MB-PSS dampens inter-area and local oscillations. The oscillations formed in the electromechanical power system; they formed disturbances in the system generator. Inter-area oscillations are produced by an oscillation among two zones in the power system. Local oscillations are generated by perturbations that appear in a power system among unstable generator and stable generator. The frequency range of local oscillations is 0.8Hz to 4.0Hz. The MB-PSS oscillation damping three frequencies scales to dampen total frequency oscillation spectrum which can appear in the power system reached. High frequency oscillation associated to local mode. Medium and low frequency oscillations associated to inter-area mode.

## Objective function

By altering the PSS blocks parameters. including the damping of electromechanical modes. the foremost formative aspect of PSS design can significantly enhance. The primary purpose of this part is to identify the best parameter values for PSS that can guarantee the system's stability under varying perturbations and effectively dampen rotor oscillations. as demonstrated in this study. By optimizing the PSS parameters. the excitation system can be controlled using AVR to dampen small signal oscillations. The CNBA algorithm objective has been progressively applied to maximize the damping ratio value given by the OF. The optimization problem of this study is formulated as follows by Eqs. (5), (6):

5 $$OF({K}_{m}.{T}_{1m}.{T}_{3m})=max(min({\xi }_{i}))$$

6 $$\left\{\begin{array}{l}{\lambda }_{i}={\sigma }_{i}\pm j{\omega }_{i}\\ {\xi }_{i}=\frac{-{\sigma }_{i}}{\sqrt{{{\sigma }_{i}}^{2}+{{\omega }_{i}}^{2}}}\end{array}\right.$$

where ${\lambda }_{i}$ and ${\xi }_{i}$ are the eigenvalues and damping ratios of ${i}^{th}$ mode respectively. $\sigma$ is the system real part of the poles and $\omega$ is the pulse oscillation.

Subject to the constraint that PSS must cover the electromechanical oscillations frequency in the limit of 0.1–3 Hz. It should be emphasized that the PSS parameters are usually limited. As a result. in favor of each PSS. three parameters a gain with two time constants must be optimized here under the following constraints in Eq. (7):

7 $$\left\{\begin{array}{l}1\,\,\,\,\,\,\,\le {K}_{m}\le 80\\ 0.01\le {T}_{1m}\le 1\\ 0.01\le {T}_{3m}\le 1\end{array}\right.$$

where $m$ is PSS index corresponding to the $m$ generator of the system. In this study. the other parameters (${T}_{W}.{T}_{2m}$. ${T}_{4m}$) of PSS are considered constants in a way that ${T}_{W}=10$. ${T}_{2m}=0.1$ and ${T}_{4m}=0.05$ to reduce the time computation and cover the mentioned oscillations as well as to limit the search space for avoiding worst fitness value by decreasing the optimized parameters.

## Proposed algorithm

### Bat algorithm

The Bat algorithm (BA). a metaheuristic algorithm inspired by the echolocation of microbats. was developed by Xin She Yang^37^ Echolocation in nature occurs over very short time periods. ranging from a few thousandths of a second to around 8–10 ms. with a variable frequency between 25 and 150 kHz. These frequencies correspond to wavelengths of 2–14 mm in the air^73^. Microbats employ echolocation to locate prey during their foraging. They emit short pulses while flying. but their pulse rate and frequency increase when they detect a potential prey. This tuning of frequency. along with the increased pulse emission rate. shortens the echolocations wavelength. resulting in better detection accuracy^41,74^. These echolocation behaviors of microbats can be generalized as the following guidelines:

- All bats employ echolocation to sense distance. and they are able to distinguish between prey/food and background obstacles in a remarkable way.
- The bats move in a random fashion with velocity ${\text{v}}_{\text{j}}$ at position ${\text{x}}_{\text{j}}$. with an unvarying frequency ${f}_{min}$. and varying loudness ${\text{A}}_{0}$ and wavelength $\uplambda$ in search of prey. Regarding to their target proximity. they can tune the rate of pulse emission $\text{r }\in [0. 1]$ and alter the wavelength (or frequency) of their emitted pulses.
- While the loudness can vary in several ways. we assume the variation of the loudness from a high initial value ${\text{A}}_{0}$ to a lowest constant value ${\text{A}}_{\text{min}}$;

The velocity and position of each bat ($\text{j}$) in a d-dimensional search space are denoted as ${\text{v}}_{\text{j}}$ and ${\text{x}}_{\text{j}}$. respectively. The updated velocity and position at time step $t$. ${v}_{\text{j}}^{t}$ and ${x}_{\text{j}}^{t}$. for generating novel solutions are expressed in Eqs. (8)–(10) as next:

8 $${f}_{\text{j}}={f}_{min}+({f}_{max}-{f}_{min})\times \alpha$$

9 $${v}_{\text{j}}^{t}={v}_{\text{j}}^{t-1}+({x}_{\text{j}}^{t-1}-{x}^{*}){\times f}_{\text{j}}$$

10 $${x}_{\text{j}}^{t}={x}_{\text{j}}^{t-1}+{v}_{\text{j}}^{t}$$

The current global optimal location is represented by ${x}^{*}$. and obtained at the current iteration by comparing the entire solutions surrounded by all $n$ bats. Here. $\alpha$ represents a vector of random drawn from a uniform distribution in the bounds of [0.1]. The velocity increment is obtained through the ${\lambda }_{\text{j}}$ and ${f}_{\text{j}}$ product. To adjust the velocity change. one can fix either ${f}_{\text{j}}$ (or ${\lambda }_{\text{j}}$) and tune the other factor accordingly. To implement the algorithm. each bat is related to a frequency randomly drawn from a uniform distribution between ${f}_{min}$ and ${f}_{max}$. The local search involves a random walk in the region of the present optimal solutions. and using the following method. a new solution intended for each bat expressed in Eq. (11) can be locally generated.

11 $${x}_{new}={x}_{old}+\varepsilon {A}^{t}$$

One can express the average loudness of all bats at a particular time step as ${\text{A}}^{\text{t}}=<{\text{A}}_{\text{j}}^{\text{t}}>$. where $\upvarepsilon$ represents a random number in the bound of [0.1]. Since the loudness of a bat usually decreases after it has caught its prey. while the rate of pulse emission increases. the appropriate value for loudness can be selected. One common way to choose the loudness is from the range of ${[\text{A}}^{0}.{A}_{min}]=[1. 0]$. where ${\text{A}}_{\text{min}}=0$ indicates that a bat has already caught its prey and stopped emitting any noise. The loudness and pulse emission rate are expressed by Eq. (12):

12 $${r}_{\text{j}}^{t+1}={r}_{\text{j}}^{0}\left[1-\text{exp}\left(-\gamma t\right)\right]. {A}_{\text{j}}^{t+1}=\beta {A}_{\text{j}}^{t}$$

For any $\upgamma$ greater than 0 and a value of $\upbeta$ between 0 and 1. where $\upbeta$ and $\upgamma$ are constants. the simulated annealing method considers $\upbeta$ as analogous to the cooling factor used in a cooling schedule.

13 $${\text{A}}_{\text{j}}^{\text{t}}\to 0. \;\; {\text{r}}_{\text{j}}^{\text{t}}\to {\text{r}}_{\text{j}}^{0}. \;\; \quad \text{as} \quad \text{t}\to \infty$$

In the simplest scenario. we can choose $\upbeta$ and $\upgamma$ to be the same value. In the standard Bat algorithm. $\upbeta$ and $\upgamma$ are usually chosen to be between 0.9 and 0.975.

### Novel Bat algorithm

The extension of the standard Bat algorithm is named the NBA. incorporating the following operators^75^:

#### Habitat select

The selection of the bats' habitat is influenced by various random phenomena and is modeled as a stochastic decision for ease of representation. The threshold of the selection is denoted by P ∈ [0.1]. If the random number R between [0. 1]. is minor against P. bats have to select the quantum behavior to forage in a wide habitats range; then. in order to forage in limited habitats. bats have to select the mechanical behavior.

#### Bats with quantum behavior

The virtual bats with quantum behavior are capable of foraging in a broad limit of habitats. If the food location is found by one individual. others they should found forage from them as soon as. Therefore, in the bats swarm the global best position could be offered as the attractor. As a result, their positions can be defined by Eq. (14):

14 $${x}_{i. j}^{t+1}=\left\{\begin{array}{l}{g}_{ j}^{t}+\theta \times \left|{mean}_{ j}^{t}-{x}_{i. j}^{t}\right|\times ln\left(1/{u}_{i.j}\right)\;\; \forall \;\; {rand}_{i}\left(0. 1\right)<0.5\\ {g}_{ j}^{t}-\theta \times \left|{mean}_{ j}^{t}-{x}_{i. j}^{t}\right|\times ln\left(1/{u}_{i.j}\right) \;\; otherwise\end{array}\right.$$

#### Bats with mechanical behavior

In view of the Doppler Effect. at time step *t* the formulations of the updating offspring are considerably distinct from the corresponding portions in the basic BA.

Firstly. the frequency formula comprises of three divisions. Excepting the random chose from the value in [${f}_{min}$ ${f}_{max}$] the frequency correspondingly depends on the bats’ compensation rates for the Doppler Effect and Doppler Effect. The bats attempt to get nearer to the prey; whereas the prey attempts its best to escape from front of the bats. Now. the ${g}_{j}^{t}$ (global best solution) could be viewed the same as the prey. Additionally. while the velocity increment is the product ${{\lambda }_{i}f}_{i}$. the suitable frequency ${f}_{i}$ may meaningfully impacts whether the bats may get nearer to the prey. The bats recompense adaptively in echoes for the Doppler Effect. If ${x}_{i. j}^{t}$ is minor to ${g}_{j}^{t}$. explicitly the bats are next to the prey. the bat flies forward. The bats could absolutely recompense in echoes for the Doppler Effect. Therefore. ${x}_{i. j}^{t}$ could approach ${g}_{j}^{t}$ and the bat could catch up the prey. Then. if ${x}_{i. j}^{t}$ is superior to ${g}_{j}^{t}$. the bats could negatively recompense in echoes for the Doppler Effect. Hence. the bats could slow down and catch up the prey. The rates of compensation $c$. it adjusts with individuals.

Secondly. while for the bats’ movements. it is faintly different from the corresponding division in the basic BA. A supplementary parameter $w$ called inertia weight is added to update velocity. It is used to control the rate of inheriting the previous velocity of an individual. As described before. the NBA has other structures and gives an improvement to BA. Hence, Equations (8)–(10) can be rewritten with NBA as follows:

15 $${f}_{i}={f}_{min}+\left({f}_{max}-{f}_{min}\right)\times \alpha$$

16 $${f}_{i.j}=\frac{(c+{v}_{i.j}^{t})}{c+{v}_{g.j}^{t}}\times {f}_{i.j}\times \left(1+{C}_{i}\times \frac{({g}_{j}^{t}-{x}_{i.j}^{t})}{|{g}_{j}^{t}-{x}_{i.j}^{t}|+\varepsilon }\right)$$

17 $${v}_{i. j}^{t+1}=w\times {v}_{i. j}^{t}\times \left({g}_{j}^{t}-{x}_{i. j}^{t}\right)\times {f}_{i.j}$$

18 $${x}_{i. j}^{t+1}={x}_{i. j}^{t}+{v}_{i. j}^{t}$$

where $w\in \left[0. 1\right]$. $\varepsilon$ is the smallest constant. $C\in \left[0. 1\right]$. $c=340\text{ m}/\text{s}$ is the speed in the air and ${v}_{g.j}^{t}$ is the speed corresponding to the global best position.

#### Local search

Bats would augment the pulse emission rate and reduce the loudness. Therefore. they would silently come close to the prey. The loudness is becoming the more probable the bat may capture the prey. Furthermore. the loudness caused by the bats themselves and other objects in nature may be also taken into account. For uncomplicatedness. the relative loudness between the mean loudness of all bats and a specific bat loudness is viewed as an impact factor when bats search locally near the prey. The new position is produced locally for each bat in Eq. (19) as follows;

19 $$\left\{\begin{array}{l}if (rand(0.1)>{r}_{i}\\ {x}_{i.j}^{t+1}={g}_{j}^{t}\times (1+randn(0.{ \sigma }^{2}))\\ {\sigma }^{2}=|{A}_{i}^{t}-{A}_{mean}^{t}|+\varepsilon \\ endif\end{array}\right.$$

The gaussian distribution with a mean of 0 and standard deviation of ${\sigma }^{ 2}$ is presented by $randn\left(0.{ \sigma }^{ 2}\right)$

### Chaotic maps

An effective technique for addressing early convergence and adjusting static parameters in metaheuristic algorithms is the utilization of CMs. Recent literature on optimization has shown a growing interest in this field. making it a rapidly emerging research area. To prevent getting stuck in a local optimum for the duration of the optimization process. we have integrated chaos into the original NBA. Thus. the proposed method in this study is a hybrid approach called Chaotic NBA (CNBA). which combines several chaotic sequences with the original Bat algorithm (NBA) to improve global convergence and prevent local convergence. The mentioned CMs are chosen for their high performance in selecting the optimal solution and providing good algorithm behavior for governing the optimization problem. The most important advantage of the CM is the smooth transition between exploitation and exploration that the algorithm driven by means of CMs contains. Similarly. in this work. the authors have used CM to reintroduce initial random parameters that regularly redistribute the search space. in contrast to the standard algorithm search through self-distributions. Briefly. the following equations describe the different types of CMs.

Four CM types among many kinds of CMs are attempted in this current study^76–79^: (i) iterative map. (ii) Gaussian map. (iii) logistic map. and (iv) piecewise map. The iterative map with infinite collapses can be written as described in Eq. (20). However. the Gaussian map can be given in Eq. (21). In addition. logistic and piecewise linear map are defined as depicted in Eqs. (22) and (23). respectively.

20 $${x}_{k+1}=sin\left(\gamma \pi /{x}_{k}\right)$$

21 $${x}_{k+1}=\left\{\begin{array}{l}0\,\,\,\,\,\,\,\,\,\,\,\,\,\,\,\,\,\,\,\,\,\,{ \forall x}_{k}=0\\ \frac{1}{{ x}_{k}.mod(1)},Otherwise\end{array}\right.$$

22 $${x}_{k+1}=a{.x}_{k}\left(1-{x}_{k}\right)$$

23 $${x}_{k+1}=\left\{\begin{array}{l}\frac{{x}_{k}}{P} 0\le {x}_{k}<P\\ \frac{{x}_{k}-P}{0.5-P} P\le {x}_{k}<1/2\\ \frac{1-P-{x}_{k}}{0.5-P} 1/2\le {x}_{k}<1-P\\ \frac{1-{x}_{k}}{P} 1-P\le {x}_{k}<1\end{array}\right.$$

where $\gamma$ = 4 is an appropriate parameter. $a$ is a favorable variable between 0 and 1 and $P$ is a control variable in the range of [0. 0.5] and ${x}_{k}$ has a value between 0 and 1.

### Chaotic novel Bat algorithm

In order to achieve the aforementioned goal. we have utilized CMs techniques to adjust three parameters in the standard NBA: the random of initial frequency. the compensation rate. and the probability of habitat selection. The approach involves completely replacing the frequency and initial frequency with the aforementioned CMs. which significantly contribute to the velocity. However. it is very imperative to note that the improper application of CMs can have a negative impact on the primary algorithm and could potentially invalidate the claims made above by disrupting the solution. To address the issue of being trapped in local optima during computation. the authors have combined NBA with CMs. as described in Table 2.

**Table 2:**
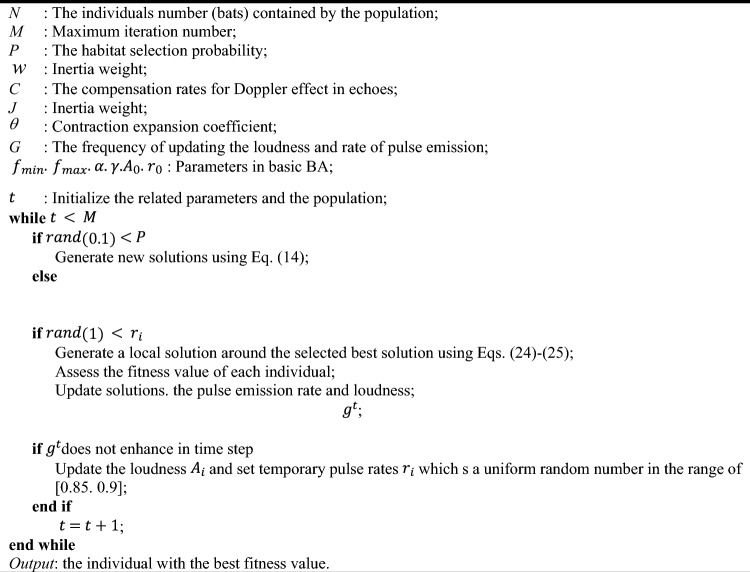
Pseudo code of CNBA based PSS.

The parameter of the initial frequency equation has been replaced by CMs. and Eq. (15) has been modified as follows:

24 $${f}_{i}={f}_{min}+\left({f}_{max}-{f}_{min}\right)\times CM$$

Also. the compensation rates of frequency equation is replaced by CM. Equation (16) is rewritten as follows:

25 $${f}_{i.j}=\frac{(c+{v}_{i.j}^{t})}{c+{v}_{g.j}^{t}}\times {f}_{i.j}\times \left(1+C{M}_{i}\times \frac{({g}_{j}^{t}-{x}_{i.j}^{t})}{|{g}_{j}^{t}-{x}_{i.j}^{t}|+\varepsilon }\right)$$

In the conventional NBA. α and ρ in the range of [0. 1] are a random number and ${c}_{i}\in \left[0. 1\right]$. Here in the developed CNBA. they have been selected as chaotic numbers between 0 and 1.

**Figure 4:**
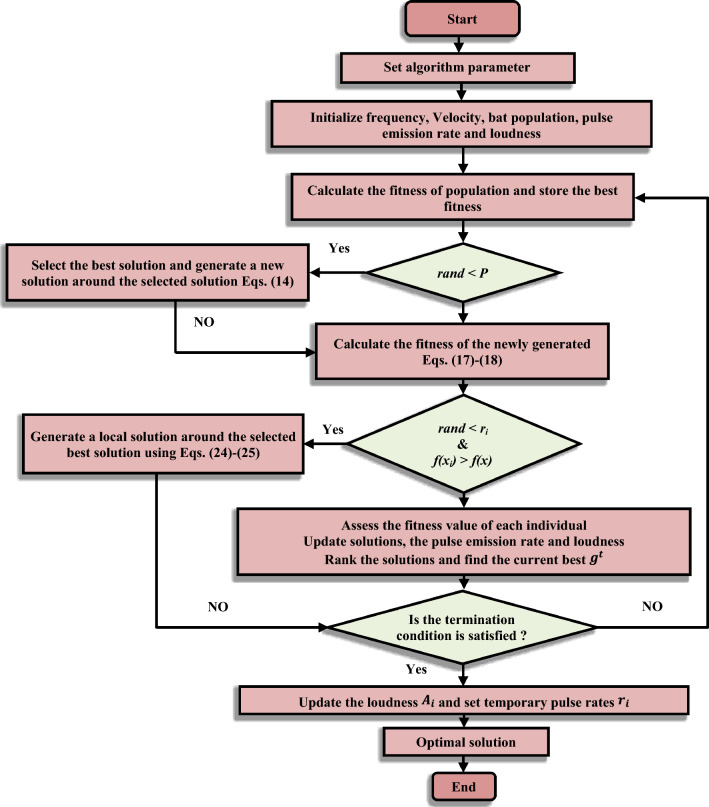
Flowchart of the CNBA based PSS.

Table 2 depicts the Pseudo code of CNBA based PSS. Figure 4 denotes the flowchart of the CNBA based PSS. The main challenge is the query response to the question of whether CNBA can be utilized to intentionally produce effects on power system stability. The response is consecutively assigned in the succeeding parts.

## Results and discussions

In this section of the paper. we briefly investigate the effectiveness of the proposed chaotic map with the NBA approach by utilizing benchmark functions outlined in^75^. Tables 3 and 4 depict the description of unimodal benchmark functions and multimodal benchmark functions. respectively.

**Table 3:** Description of unimodal benchmark functions.

ID	Formula	Range
_F1_	$f(x) = \sum\nolimits_{i = 1}^{n} {x_{i}^{2} }$	[100, 100]
_F2_	$f(x) = \sum\nolimits_{i = 1}^{n} {\left| {x_{i} } \right|} + \prod\nolimits_{i = 1}^{n} {\left| {x_{i} } \right|}$	[10, 10]
_F3_	$f(x) = \sum\nolimits_{i = 1}^{n} {\left( {\sum\nolimits_{j - 1}^{i} {x_{j} } } \right)^{2} }$	[100, 100]
_F4_	$f(x) = \max_{i} \left\{ {\left| {x_{i} } \right|,\,1 \le i \le n} \right\}$	[100, 100]
_F5_	$f(x) = \sum\nolimits_{i = 1}^{n - 1} {\left[ {100\left( {x_{i + 1} - x_{i}^{2} } \right)^{2} + (x_{i} - 1)^{2} } \right]}$	[30, 30]
_F6_	$f(x) = \sum\nolimits_{i = 1}^{n} {\left( {\left[ {x_{i} + 0.5} \right]} \right)^{2} }$	[100, 100]
_F7_	$f(x) = \sum\nolimits_{i = 1}^{n} {ix_{i}^{4} + random(0,1)}$	[1.28, 1.28]

**Table 4:** Description of multimodal benchmark functions.

ID	Formula	Range
_F8_	$f(x)={\sum }_{i=1}^{n}-{x}_{i}\mathit{sin}(\sqrt{\left|{x}_{i}\right|})$	[− 500, 500]
_F9_	$f(x)={\sum }_{i=1}^{n}\left[{x}_{i}^{2}-10\mathit{cos}\left(2\pi {x}_{i}\right)+10\right]$	[− 5.12, 5.12]
_F10_	$f(x)=-20exp(-0.2\sqrt{\frac{1}{n}\sum_{i=0}^{n}{x}_{i}^{2})}- exp(\frac{1}{n}\sum_{i=1}^{n}\mathit{cos}(2\pi {x}_{i}))+20+e$	[− 30, 30]
_F11_	$f(x)=\frac{1}{4000}{\sum }_{i=1}^{n}{x}_{i}^{2}-{\prod }_{i=1}^{n}\mathit{cos}(\frac{{x}_{i}}{\sqrt{i}})+1$	[− 600, 600]
_F12_	$f(x)=\frac{\pi }{n}\left\{10\mathit{sin}(\pi {y}_{1})+\sum_{i=1}^{n-1}{({y}_{i}-1)}^{2}[1+10{sin}^{2}(\pi {y}_{i+1})]{({y}_{n}-1)}^{2}\right\}+\sum_{i=1}^{n}u\left({x}_{i}.\text{10.100.4}\right). where {y}_{i}=1+\frac{{x}_{i}+1}{4}$ $$u({x}_{i}.a.k.m)=\left\{\begin{array}{c}k{({x}_{i}-a)}^{m}{x}_{i}>a\\ 0 -a<{x}_{i}<a\\ k{({-x}_{i}-a)}^{m}{x}_{i}<-a\end{array}\right.$$	[− 50, 50]
_F13_	$f(x)=0.1{sin}^{2}(3\pi {x}_{1})+{\sum }_{i=1}^{n}{({x}_{i}-1)}^{2}[1+{sin}^{2}(3\pi {x}_{i}+1)]+{({x}_{n}-1)}^{2}[1+{sin}^{2}(2\pi {x}_{n})]+\sum_{i=1}^{n}u({x}_{i}.\text{5.100.4})$	[− 50, 50]

Table 5 displays the results obtained subsequent to 30 runs of each algorithm for the benchmark functions. Compared to the original NBA, it is evident from Table 5, that the results obtained with CNBA outperformed the other algorithms.

**Table 5:** Statistical results of various algorithms applied on the unconstrained problems.

Fun no.	Measure	Comparative algorithms				
		NBA	NBA-Gauss	NBA-iterative	NBA-logistic	NBA-piecewise
F1	Worst	3.0896E−15	5.9477E−15	9.9171E−16	5.9921E−15	8.2602E−15
	Mean	7.5161E−16	1.1571E−15	2.1883E−16	1.0939E−15	1.7106E−15
	Best	4.5747E−26	7.1542E−25	1.4112E−28	1.5828E−24	4.1346E−23
F2	Worst	3.1233E−80	9.5413E−82	3.9725E−83	2.9107E−83	1.7048E−76
	Mean	5.7023E−81	1.7393E−82	7.2930E−84	5.6443E−84	3.1126E−77
	Best	1.0227E−99	9.2948E−100	0.0000E+00	0.0000E+00	0.0000E+00
F3	Worst	6.9712E−162	4.5921E−166	5.5137E−164	1.4215E−160	2.6984E−167
	Mean	0.0000E+00	0.0000E+00	0.0000E+00	2.6017E−161	0.0000E+00
	Best	1.1061E−195	0.0000E+00	0.0000E+00	1.9779E−198	6.8532E−196
F4	Worst	5.6880E−81	1.1198E−81	1.4598E−83	1.4071E−79	3.8166E−83
	Mean	1.1273E−81	2.0434E−82	3.0906E−84	2.5686E−80	6.9828E−84
	Best	1.1435E−98	3.1644E−98	4.1021E−107	2.3071E−98	0.0000E+00
F5	Worst	2.8733E+01	2.8731E+01	2.8741E+01	2.8746E+01	2.8718E+01
	Mean	7.1214E−01	7.8453E−01	7.7605E−01	7.2890E−01	6.4200E−01
	Best	2.6187E+01	2.6185E+01	2.5504E+01	2.5708E+01	2.6606E+01
F6	Worst	1.4921E+00	1.6150E+00	1.9878E+00	1.6906E+00	1.5137E+00
	Mean	3.8118E−01	3.5324E−01	4.5624E−01	3.8167E−01	2.9284E−01
	Best	2.7450E−01	2.2366E−01	1.7432E−01	2.3977E−01	3.4418E−01
F7	Worst	1.0228E−02	6.4382E−03	6.0443E−03	9.4781E−03	7.7133E−03
	Mean	2.3005E−03	1.5525E−03	1.4245E−03	2.0616E−03	1.5844E−03
	Best	4.7807E−04	3.5518E−04	3.0413E−04	4.3365E−04	5.5393E−04
F8	Worst	− 5.4177E+03	− 5.6561E+03	− 5.6561E+03	− 5.6561E+03	− 5.6561E+03
	Mean	9.7638E+02	8.8420E+02	9.7017E+02	8.5101E+02	9.1492E+02
	Best	− 8.9265E+03	− 8.4479E+03	− 8.0456E+03	− 8.3091E+03	− 8.8559E+03
F9	Worst	2.4236E+02	2.3231E+02	2.2293E+02	2.6749E+02	2.4733E+02
	Mean	4.7470E+01	4.7994E+01	4.3035E+01	4.8984E+01	4.7507E+01
	Best	7.8105E+01	3.7229E+01	2.4284E+01	6.3807E+01	6.8736E+01
F10	Worst	1.0594E−04	5.1241E−05	3.7903E−05	6.9459E−05	4.4439E−05
	Mean	2.7045E−05	1.0798E−05	9.2027E−06	1.4019E−05	1.0362E−05
	Best	6.1365E−06	2.4973E−06	1.9148E−06	2.0474E−06	2.7851E−06

**Figure 5:**
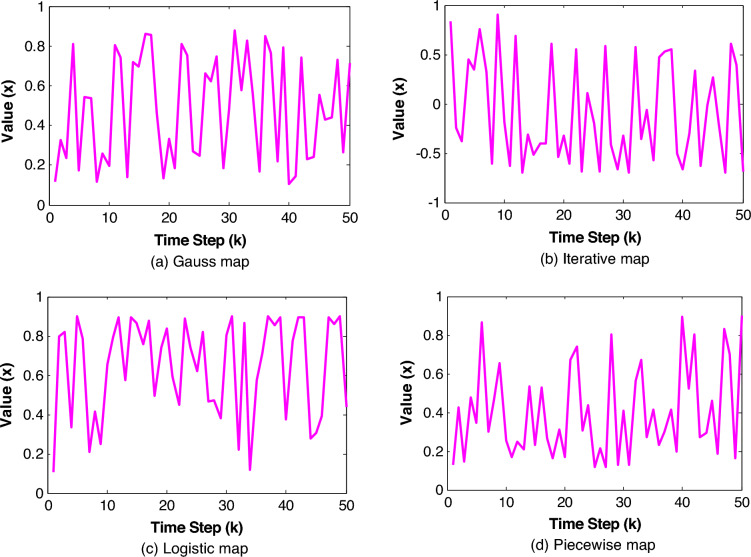
Distributions of chaotic value.

Among the CM used at this point. in case of solving unconstrained global optimization problems. Iterative map notably attained better solution quality and can boost search strength in the designed trouble space. These results confirm the proposed approaches applicability that offer the aptitude to accomplish the global optimum for the tested functions. The preeminence of this map to boost the diversification and intensification operations is demonstrated by its prosperity in swiftly achieving a balance between local and global search to reach the exact solution. As a result. we have opted to use the iterative map for addressing the problem of power system stability in the following parts. As can be observed. other CM can absolutely boost the competence of NBA with the exception of the Piecewise map where the attainment of the global minimum appears to be unachievable in the case of the Step function and Logistic map with Rastrigin function. Figure 5a–d illustrates the distributions of chaotic value of 50 generations for different maps with random initial values.

To assess the robustness of the approaches. there are several criteria amid them the success rate. At this point; we have applied the success rate criterion that can be defined in Eq. (26) as follows:

26 $${S}_{r}=100\times \frac{{N}_{successful}}{{N}_{all}}$$

where ${N}_{all}$ is the runs number. and ${N}_{successful}$ is the run number in which the solution is successful.

**Figure 6:**
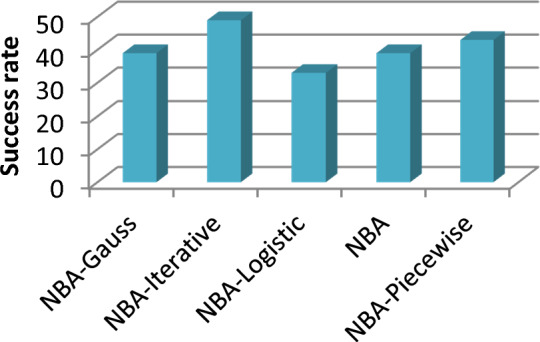
Success rate of Rastrigin function.

In this study. the authors selected a run to be successful when the obtained solution is very close to the global optimum. The audience can observe from success rates visualized in Fig. 6 the suggested approach can generally improve the results quality. and they attained the reliability of the global solution.

The subsequent study treated different aspects of our problem so that it is divided in two main subsections:

### Optimization of PSS parameters

This section explores an improvement to NBA through the use of CMs. which enhances the search strategy for the duration of the optimization process and facilitates the identification of optimal parameters for PSS design. The power system performance is taken into consideration during this process. This actual plan is tendered to construct an excellent damping for inter-area and local oscillations hence should be withstood diverse operating cases then also default defects. The optimization technique is employed here as an important task and alone to control the stabilizers. To authenticate the competent performance of the proposed tuning algorithm. a well-known power system which is 16-machine. 68-bus has been simulated as a suitable system for dynamic stability studies based on various extremity contingencies via nonlinear and state space representations. The PSS parameters should be optimized are; the PSS gain ($K$) and lead-lag time constants (${T}_{1}$ and ${T}_{3}$). There are 48 variables of control to be adjusted in the test system.

It is worthy stating that the values of ${A}_{0}$, ${r}_{0}$, $P$, $w$, $C$, and $\theta$ are dynamically calculated from the stipulated min/max values specified in Table 6. The formulas used to realize such dynamic calculations are depicted in Eqs. (27)–(32); respectively.

27 $${A}_{0 }=rand\left(\dots \right)\times \left({{A}_{0 }}_{max}-{{A}_{0 }}_{min}\right)+{{A}_{0 }}_{min}$$

28 $${r}_{0}=rand\left(\dots \right)\times \left({{ r}_{0}}_{max}-{{ r}_{0}}_{min}\right)+{{ r}_{0}}_{min}$$

29 $$P=rand\left(\dots \right)\times \left({P}_{max}-{P}_{min}\right)+{P}_{min}$$

30 $$w=\frac{t-G}{t}\times \left({w}_{max}-{w}_{min}\right)+{w}_{min}$$

31 $$C=rand\left(..\right)\times \left({C}_{max}-{C}_{min}\right)+{C}_{min}$$

32 $$\theta =\frac{t-G}{t}\times \left({\theta }_{max}-{\theta }_{min}\right)+{\theta }_{min}$$

where $t$ defines iteration index and $G$ is maximum iterations.

**Table 6:** Parameters of NBA.

Algorithm	Parameters
NBA	$\alpha =\gamma =0.9$. ${f}_{min}=0. {f}_{max}=1.5$ ${A}_{0 }\in [0. 1]. { r}_{0}\in [0. 1]$*. G* = *10*
	$P\in [0.5. 0.9]. w\in [0.4. 0.9]$ $C\in [0.1. 0.9]$.$\theta \in [0.5. 1]$

**Figure 7:**
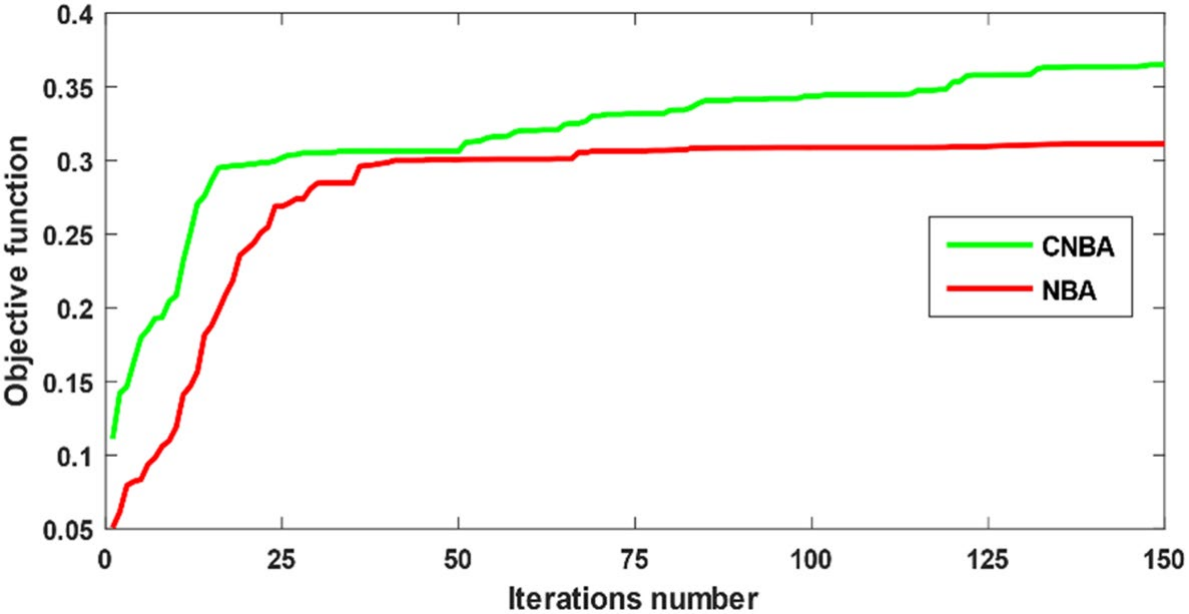
Convergence graph of the objective function for case 1.

The authors also successfully performed the similar simulation by implementing NBA algorithm to contrast and disclose the proposed CNBA effectiveness. Before starting the calculation process. some specified parameters should be set in the CNBA and NBA for acquiring reliable performance and shunning primarily immature convergence. The diagram problem capacity and complexity lead to increase maximal generations and population size for pledging optimal results taking into account control variables number. The parameters for each algorithm are specified as follows: the maximum iterations number and the number of individuals in the population are set to 150 and 96. respectively. The Convergence graph of the objective function for case 1 is illustrated in Fig. 7. The other parameters used in favor of this study are summarized in Table 5.

The preeminent results achieved with the above problem sustain us to confirm the performance of the proposed CNBA-PSS in a comprehensive system presented in “Introduction” section. Hence. a time-domain simulation has been carried out according to large disturbances in the test system. For each case. the faults are applied at 1.0 s after the simulation start and cleared at 1.09 s. To appraise the robustness of the proposed stabilizer under large disturbance; three severe perturbations are considered below. Table 5 presents the best parameters acquired by the suggested algorithms. Table 7 presents the best parameters acquired by the employed algorithms.

- Case 1: Line 9–8 is out of service. between two areas.
- Case 2: Line 1–2 is out of service. between two areas.
- Case 3: Line 4–5 is out of service. load increase 25% in bus 20. 21; generation increase 20% in G9.

**Table 7:** Optimal parameters of PSS for case 2.

Generators	NBA			CNBA		
	$K$	${T}_{1}$	${T}_{3}$	$K$	${T}_{1}$	${T}_{3}$
*G1*	21.3193	0.9981	0.9397	60.5556	0.9137	0.5062
*G2*	73.416	0.6033	0.2125	14.3432	0.398	0.9966
*G3*	79.3785	0.6033	0.32	43.3559	0.2008	0.8094
*G4*	19.5137	0.3677	0.7165	14.3863	0.4564	0.6137
*G5*	5.102	0.3653	0.042	15.6856	0.1437	0.5468
*G6*	26.0609	0.8738	0.4064	38.1728	0.8664	0.5412
*G7*	19.606	0.8848	0.1597	28.01	0.1945	0.1315
*G8*	56.1603	0.5694	0.4053	7.7604	0.8931	0.8
*G9*	19.5746	0.7308	0.0745	41.6836	0.1143	0.2151
*G10*	9.5904	0.9874	0.4744	10.3006	0.1198	0.898
*G11*	6.3072	0.133	0.5551	3.6006	0.236	0.3351
*G12*	26.9821	0.6891	0.1283	8.6258	0.0847	0.9636
*G13*	3.4082	0.9965	0.0785	17.9393	0.2305	0.7534
*G14*	38.6248	0.0257	0.9991	50.5743	0.2762	0.3327
*G15*	72.2753	0.0362	0.2457	56.1683	0.4753	0.0667
*G16*	7.3234	0.0938	0.7491	76.1502	0.4105	0.1797

**Figure 8:**
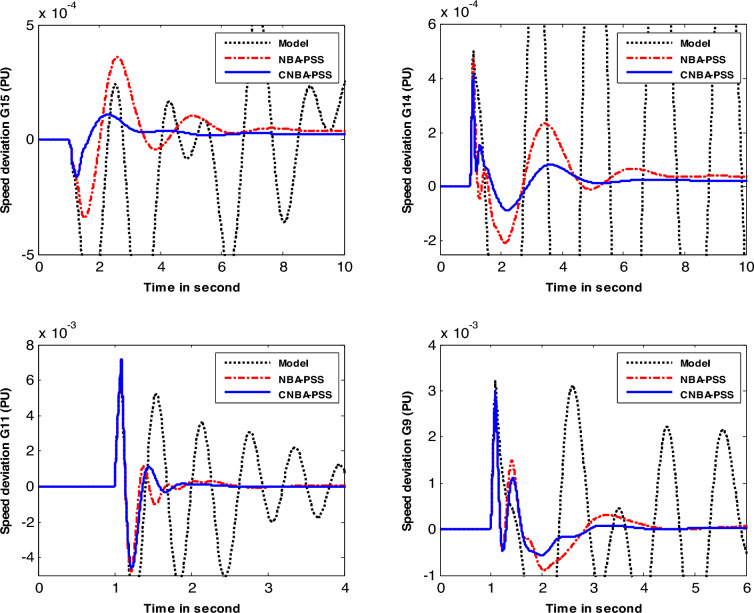
Speed deviation responses of the system for case 1.

**Figure 9:**
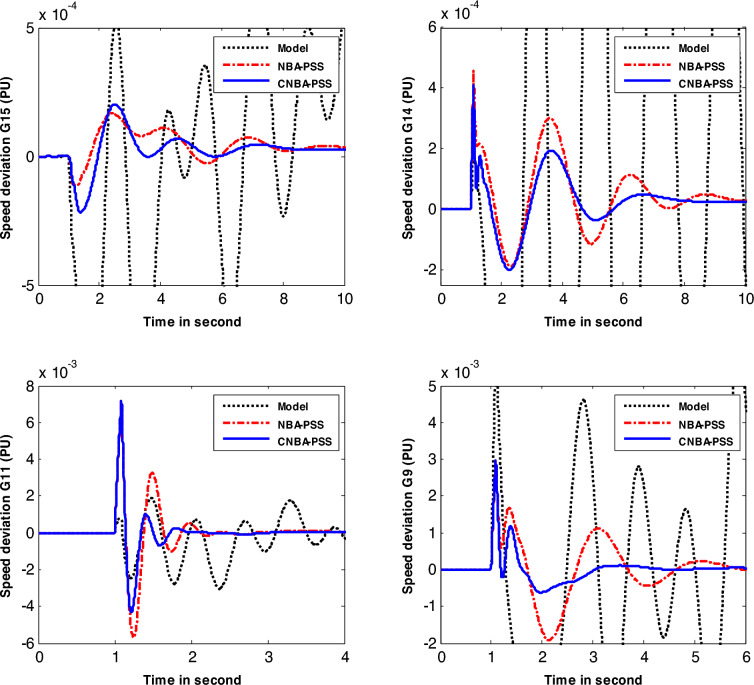
Speed deviation responses of the system for case 2.

Figures 8 and 9 show the system response of generators under the disturbance of case 1 and case 2. respectively. with various algorithms. The simulation results illustrate that the designed CNBA-PSS stabilizer provides significantly improved and robust performance for system stability. with better damping compared to the NBA. In addition. it can be vividly seen that the CNBA-PSS stabilizer gives adequate settling time ($\text{Ts}$) and overshoots for power oscillations under this severe disturbance which appears posthaste restitution of stability subsequent the perturbation and a proper damping is obtained. Equitably. NBA-PSS contributes in improving the damping of power system oscillation and furnishes a satisfactory response. Finally. these figures thoroughly validate the robustness of PSS using CNBA comparatively with another stabilizer. Better outcomes due to the ability of iterative map in the control mechanism that guarantees the equilibrium between exploitation and exploration further prevent early convergence to suboptimal PSS parameters.

**Figure 10:**
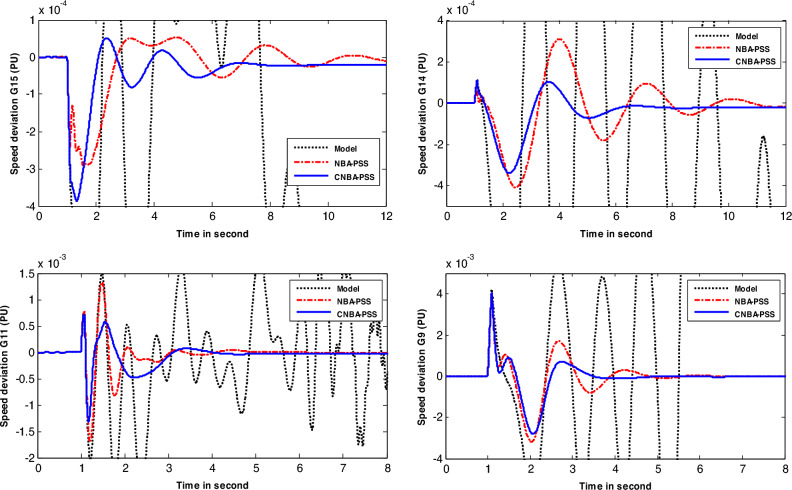
Speed deviation responses of the system for case 3.

In case 3, the authors considered the system with changing in operating condition subjected to the applied perturbation to better compare the result achieved with its counterpart in the first and second cases. The speed response of generators-based tuning for case 3 is given in Fig. 10. Based on the obtained results. the PSS designed using CNBA demonstrates superior performance particularly in terms of Ts and overshoot. Therefore. it can be inferred that the power system with the suggested stabilizer exhibits an overall superior and resilient stability performance. and offers better damping compared to another algorithm. More specifically. this yielded result proves the superiority of the suggested CNBA in PSS tuning. which proves its ability and flexibility to achieve the best choice of the parameter settings even in severe scenarios due to its computational efficiency.

**Figure 11:**
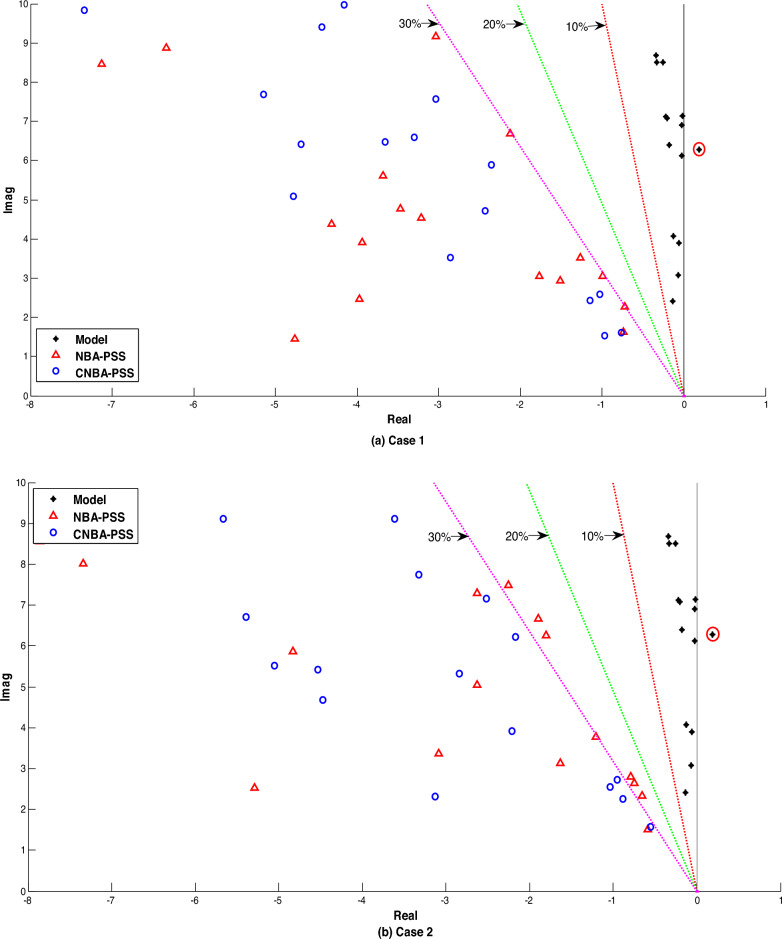

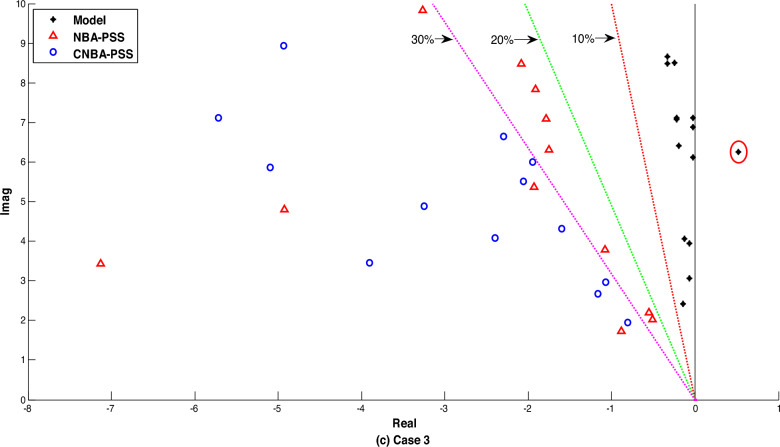
Eigenvalues distribution of electromechanical modes for cases 1. 2 and 3.

Figure 11 illustrates the system eigenvalues for the mentioned systems with the proposed PSS tuning. along with the eigenvalues of the electromechanical modes on the damping ratios axis. Overall. it can be noted from the results of the power system without installed PSS that one mode is positive and other modes are poorly damped for all cases. this referred that the system is quite unstable. Otherwise. A noticeable shift of all electromechanical modes. including local and interregional. towards the left side of the s-plane can be observed with the designed CNBA-PSS. by improving both the damping ratio and the eigenvalues real part concurrently. which can also be articulated that the system damping characteristics are greatly enhanced upwards 30% under all cases in comparing with NBA-PSS. The superior performance achieved by the linear model is in agreement with that achieved through the nonlinear model. The overall analysis of the results highlights the inherent advantages of the proposed CM in achieving optimal power system stability and mitigating the limitations of the NBA approach.

For instance. to evaluate the preciseness of system response and improvement in time-domain simulation and make comparison consistency. the performance index Integral Absolute Error (IAE) is considered and judged for statistically quantifying and analyzing the efficacy of the suggested stabilizers and is defined as:

33 $$IAE={\int }_{0}^{{t}_{sim}}\left|e\left(t\right)\right|dt$$

where ${t}_{sim}$ is total simulation time and $\left|e\right|$ symbolizes the absolute error.

Table 8 provides the values of the IAE and $\text{Ts}$ that were computed for the generators speed response included in the system study. comparing the performance of the CNBA-PSS and NBA-PSS approaches. On closer examination of the values in this table. it can be inferred that the proposed stabilizer using the CNBA algorithm attains the minimum value of IAE. Additionally. it is clear that the same algorithm-based stabilizer obtains the lowest value of $Ts$.

**Table 8:** Performance index and *Ts* values for New-England power system.

Case #	Criteria	Method	G 9	G 11	G 14	G 15
Case 1	IAE	CNBA	0.3921	0.7397	0.0797	0.0602
		NBA	0.4977	0.7891	0.1281	0.1368
	$\text{Ts}$	CNBA	3.2495	1.7451	5.4432	7.6066
		NBA	3.9975	2.4288	7.1562	8.2287
Case 2	IAE	CNBA	0.4111	0.7327	0.1170	0.0852
		NBA	0.8001	1.0518	0.1698	0.0943
	$\text{Ts}$	CNBA	3.9097	1.8805	7.5417	8.0506
		NBA	5.6403	2.6817	9.1398	10.9434
Case 3	IAE	CNBA	0.8493	0.2320	0.1178	0.1154
		NBA	0.9620	0.2606	0.1988	0.1181
	$\text{Ts}$	CNBA	3.4106	3.9783	6.9476	6.3225
		NBA	5.2838	4.7118	11.0287	11.6255

The numerical results provide clear evidence that the CNBA outperforms the NBA in terms of error and $\text{Ts}$. confirming that the CM reduces the probability of the NBA getting trapped in local minima by mutating the internal mechanism of control. These results are crucial for ensuring the stable and effective operation of the power system under various operating conditions. Consequently. the remarkable efficient signal provided by CNBA-PSS helps to limit the electromechanical oscillation.

### Optimal location of PSS

Thorough study on PSS location has been considering an involved aspect and more challenging due to the sensitivity of the stabilizers and power system. PSS can provide modest consequences exactly when its site is not well studied. Under this circumstance. the operative technique has been applied here to identify the best number and location of PSS via the PF technique and proposed algorithm. and auxiliary to scrutiny the impact of fewer stabilizers in plant efficiency.

**Figure 12:**
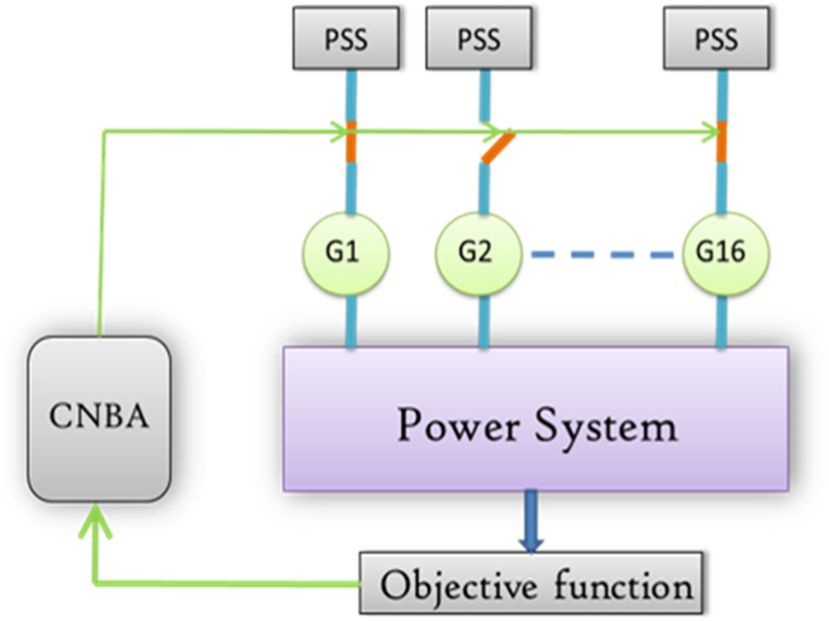
Procedure of PSS selection.

The framework of steps arrangement is mainly realized by the inverse process of PSS placement which are by equipping all generators with PSS through the above optimized parameters then the objective algorithm is employed from this tip for elimination the unproductive PSS all the way passing through severe constraint defined by the maximum damping ratio does not diminish even 7%. that is to say the OF is the PSS number as illustrated in Fig. 12. The major advantage of the PSS number lessening using CNBA is that the cost of the scheme is decreased and covered by reducing in tandem the PSS number. In addition, to attest that the proposed CNBA has further the ability for establishing simultaneously the optimal locations of the stabilizers and it is capable to make use of the required PSS number minimum. Consequently. the blunder of PSS position may hinder largely the power system reliability. The search space is quite decreased by inserting the same control variables given in “Introduction” section. In difference. it has been added some factors such as PSS location and number which contribute in objective fitness value similar to the previous part.

The proposed work can fine-tune the optimal PSS location and discard from unavailing PSS corresponding to the OF thus maintaining the dynamic stability at a specific level through a few PSS numbers. Best outcomes have been visually embedded within the strategy drawn. The suitable location of PSS is indicated in Table 9 using CNBA that is proving its effectiveness in computational complexity.

**Table 9:** Best location using CNBA.

Case 2	G1	G2	G3	G4	G6	G8
	G9	G10	G12	G14	G15	
Case 3	G1	G2	G3	G5	G7	G8
	G9	G10	G12	G14	G16	

Eigenvalue analysis. frequency. damping ratio. and mode of oscillations under case 2 have been explicitly summarized in Table 10. It is noticeable from the table that generator G7 located in bus 57 has a slight contribution in mode 2. Hence. the PSS is ineffective for positioning in this generator to damp out the inter-area oscillation. In contrast. to assess reasonable comparison. it is obligatory to install 11 PSS for large system using FP corresponding to the results obtained with CNBA. At the same manner, 5 from 16 for large system according to minimum participation have been eliminated.

**Table 10:** Participation factors of speed associated with the electromechanical modes for case 2.

Mode no	Eigen value	Freq.	D. ratio	Mode	Gen.	PF
1	− 0.1302 + 4.0683i	0.6475	0.0320	Inter-area	68 66 67	0.1133 0.2597 0.6240
2	− 0.0626 + 3.8980i	0.6204	0.0161	Inter-area	57 58 65	0.0812 0.1032 0.4071
3	− 0.0647 + 3.0679i	0.4883	0.0211	Inter-area	66 68	0.4086 0.5483
4	− 0.1381 + 2.4072i	0.3831	0.0573	Inter-area	66 67 65	0.1608 0.1946 0.3361
5	− 0.6158 + 10.4607i	1.6649	0.0588	Local	63	0.9960
6	− 0.3381 + 8.6847i	1.3822	0.0389	Local	56	0.7258
7	− 0.2575 + 8.5000i	1.3528	0.0303	Local	60	0.8994
8	− 0.3350 + 8.5017i	1.3531	0.0394	Local	59	0.6464
9	− 0.0200 + 7.1346i	1.1355	0.0028	Local	53	0.5595
10	− 0.0271 + 6.9007i	1.0983	0.0039	Local	62	0.5752
11	− 0.2225 + 7.1167i	1.1327	0.0313	Local	55	0.5321
12	− 0.2072 + 7.0845i	1.1275	0.0292	Local	57	0.4433
13	− 0.1762 + 6.4007i	1.0187	0.0275	Local	61	0.7273
14	0.1910 + 6.2814i	0.9997	− 0.0304	Local	54	0.3885
15	− 0.0223 + 6.1134i	0.9730	0.0036	Local	64	0.7695

**Figure 13:**
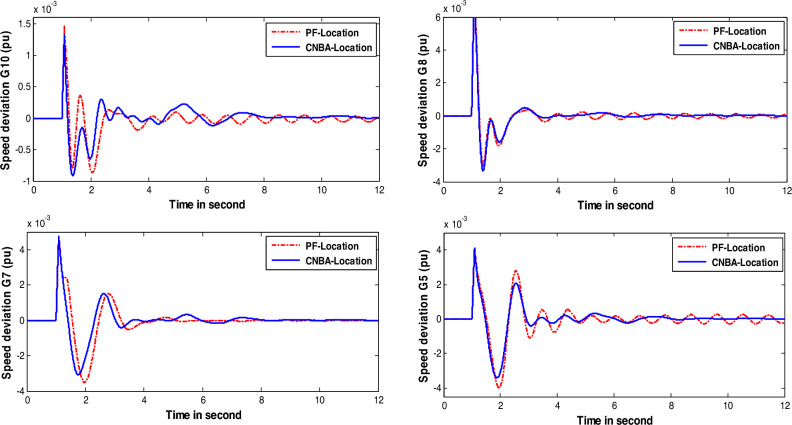
Speed deviation responses for case 2.

**Figure 14:**
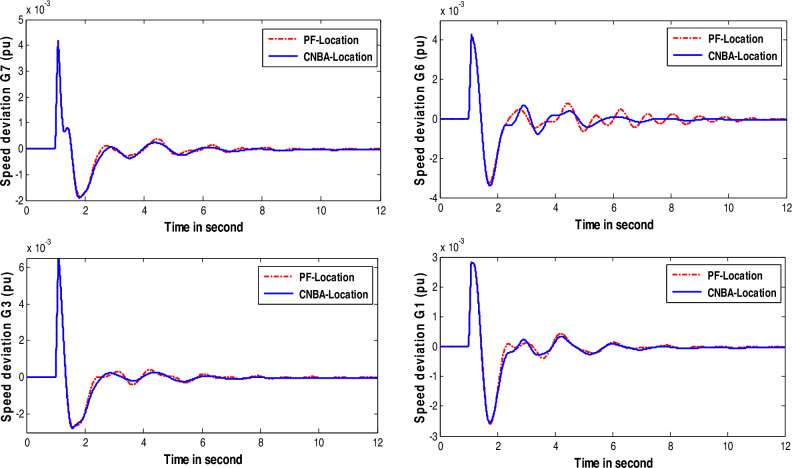
Speed deviation responses for case 3.

**Figure 15:**
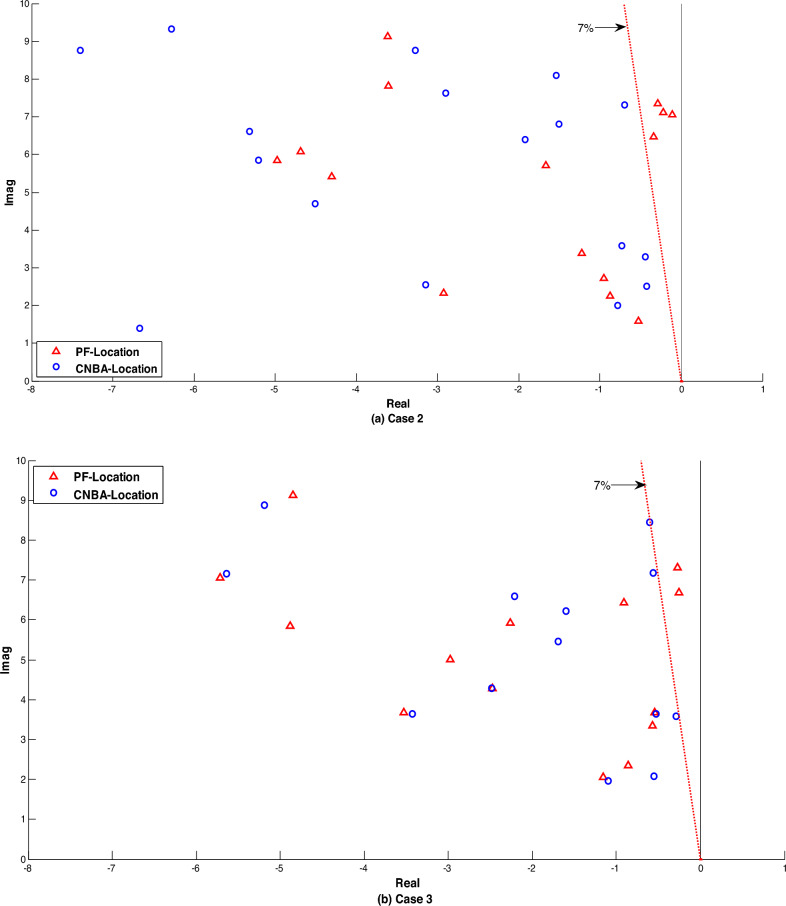
Eigenvalues distribution of electromechanical modes under case 2 and 3.

The generator response is displayed that fitted by PSS and associated with both techniques (see in Figs. 13 and 14) indicating good results. From a quick look. generators speed deviations using CNBA-PSS afford best performance and exemplified almost same peak time but lower error criterion in some generators compared to the speed deviations using PF placement due to featured repositioning of PSS as given in these figures. Indeed. the optimal PSS location using CNBA achieved best damping ratio and thus ensured best coherence and coordination between the PSS that donated high performance to the power system in comparison with the proposed PF technique. It is notable from Fig. 15. that both the inter-area and local modes of oscillations are absolutely decreased by positioning a few PSS in both the modes using CNBA rather than PF placement. Also. the overall power stability system can then be properly achieved by simultaneous interactions between effective PSS. Generically. the technique performance of PSS placement using CNBA surpassed the PF technique.

##  Conclusion

The successful application of numerous chaotic maps in addition to the conventional NBA has enhanced the optimum PSS adjustment in a multimachine power system in this paper. In order to demonstrate this proposition's capacity and applicability. the authors first applied it to unrestricted mathematical problems. The methodology utilized here was to apply the proposed CNBA to the 16 generator and 68 bus interconnected power system of New England and New York for a variety of loading circumstances and perturbations. The purpose of the paper was to compare the performance of the designed CNBA-PSS with that of the NBA-PSS using nonlinear simulation and eigenvalue analysis. as well as to determine the best PSS parameter values for achieving satisfactory oscillation damping by focusing on the damping ratio of electromechanical modes with low damping. The IAE performance index and Ts. which is the lowest value and is compared to others. are also used to analyze the CNBA-PSS performance. The second methodology offered a reliable method for using CNBA to determine the greatest objective fitness value to determine the ideal location and amount of stabilizers. The robustness of the methodology used in greatly enhancing the dynamic stability of the system is clearly demonstrated by the excellent simulation results.

CNBA algorithm can sustain from untimely convergence. which means that the method converges to a local best instead of the global best. This limitation can be mostly difficult in complex. several-modal optimization problems.

There are plans to expand the current research and analyze a broader range of chaotic maps. Furthermore. efforts will be made to apply the optimization technique to solve real-world systems. As a future endeavor. it is suggested that the validity of the findings be further confirmed through experimental tests or real-time hardware in the loop simulations.

This article focuses on numerous promising opportunities for future research with the improved NBA. It suggests expanding CNBA application to areas such as the problem of Optimal Power Flow (OPF) and the Energy Management optimization solving within Micro-grid Maximum Power Point Tracking (MPPT) for improved PV system performance. parameters identification of Proton Exchange Membrane Fuel Cells (PEMFCs) model.

## Data Availability

The datasets used and/or analysed during the current study available from the corresponding author on reasonable request.
